# Translational movement within the glenohumeral joint at different rotation velocities as seen by cine MRI

**DOI:** 10.1186/s40634-018-0124-x

**Published:** 2018-03-15

**Authors:** Kazuhisa Matsui, Takashi Tachibana, Katsuya Nobuhara, Yasushi Uchiyama

**Affiliations:** 1 Department of Physical Therapy, Graduate School of Medicine, School of Health Sciences, Nagoya University, 1-1-20 Daiko-Minami, Higashi-ku, Nagoya-shi, Aichi-ken, Nagoya City, 461-8673 Japan; 2 Department of Rehabilitation, Gifu Junior College of Health Science, 2-92 Higashi-Uzura, Gifu, 500-8281 Japan; 3 Department of Physiotherapy, Nobuhara Hospital, 720 Haze, Issai, Tatsuno, Hyogo 679-4017 Japan; 4 Institute of Biomechanics, Nobuhara Hospital, 720 Haze, Issai, Tatsuno, Hyogo 679-4017 Japan

**Keywords:** Dynamic glenohumeral stability, Intraarticular movement, Rotation velocity, Cine MRI

## Abstract

**Background:**

The glenohumeral joint is subjected to opposing forces when the direction of shoulder motion is changed, accelerating and decelerating to make the movements. The influence of motion velocity or acceleration on translation of the humeral head has not been evaluated although direction and distance of humeral head translation has been analyzed in real time in normal shoulders. We hypothesized that, in a normal shoulder, the humeral head does not deviate significantly or suddenly during active shoulder rotation regardless of motion velocity. The purpose of this study was to clarify normal intraarticular kinematics of humeral head position and translation during axial shoulder rotation with the arm by the side of the body at different rotational velocities using cine magnetic resonance imaging (MRI).

**Methods:**

Both shoulders of ten healthy adults (mean age group between 27.80 ± 6.05 years) were used in this study. Prior to MRI scan, dynamic glenohumeral stability was confirmed by physical examination. The glenohumeral joint was scanned during active shoulder rotation at three angular velocities (low, medium and high velocities), with the arm by the side of the body by real-time cine MRI while recording with the help of a video camera. Translation of the humeral head and rotation angles on MR imaging and video camera were measured to match shoulder rotational positions.

**Results:**

There were no statistical differences of the humeral head position and translation among three rotation velocities (*p* > 0.05). Translation of the humeral head was distributed from 1.44 ± 2.45 mm anteriorly to 0.65 ± 1.84 mm posteriorly at low velocity, from 0.74 ± 1.92 mm anteriorly to 0.75 ± 2.17 mm posteriorly at medium velocity, and from 2.62 ± 2.19 anteriorly to 1.17 ± 1.44 mm posteriorly at high velocity.

**Conclusions:**

Translation of the humeral head was shown to undergo no significant change throughout the ranges of internal and external rotation, or among different rotational velocities in dynamic stability of the glenohumeral joint.

## Background

The glenohumeral joint is a morphologically unstable joint, which articulates the large surface of the humeral head with the shallow and small surface of the glenoid fossa. Hence, dynamic stability of the glenohumeral joint requires maintaining the humeral head on the glenoid fossa by appropriate onsets of rotator cuff muscles and deltoid muscles (Favre et al. 2012) and by the resulting compressing force towards the centre of the glenoid fossa (Lippitt and Matsen 1993) during active shoulder motion. In contrast to the structural stability, the effect of muscle contraction is essential in dynamic stability. An in vivo study demonstrated that muscle contraction influenced the humeral head at static rotational positions (Robert-lachaine et al. 2015). This study revealed that evaluation of intraarticular movement during active movement is vital in the assessment of dynamic stability.

Coordinated muscle control of the glenohumeral joint is evaluated through findings of deviation of the humeral head and of the rotation axis during active shoulder rotation at various positions in the different types of contraction patterns and motion velocities (Magarey and Jones 2003). Translation of the humeral head during shoulder rotation has provided quantitative evidence in vivo studies (Bey et al. 2008; Dal Maso et al. 2014, 2015; Kozono et al. 2017). Posterior translation was observed at the end of internal rotation in a biplane radiographic study (Bey et al. 2008). More than 3 mm translation in the antero-posterior direction was reported in motion capture studies (Dal Maso et al. 2014, 2015). Those studies provided data on humeral translation during external rotation only (Bey et al. 2008; Kozono et al. 2017). The translation of the humeral head during internal rotation has not been reported.

The glenohumeral joint undergoes a wide range of motion at various speeds in day-to-day activities, at work, and in sports. The glenohumeral joint is subjected to opposing forces when the direction of shoulder motion is changed, accelerating and decelerating to make the movements. Physical evaluation for coordinated muscle control thus involves various motion velocities (Magarey and Jones 2003). Such evaluation is vital as altered muscle onset timing has been revealed in the unstable shoulder (Rajaratnam et al. 2013). Previous studies analyzed translation of the humeral head during active external rotation, with various motion velocities ranging from 27.5°/s to 32.5°/s (Bey et al. 2008; Kozono et al. 2017). Several imaging studies pertaining to variation of motion velocities to analyze glenohumeral intraarticular movements have not revealed consistent findings (Bey et al. 2008; Kozono et al. 2017). To our knowledge, the effect of varying motion velocity or acceleration on translation of the humeral head has not been evaluated although direction and distance of humeral head translation has been analyzed in real time in normal shoulders (Bey et al. 2008; Kozono et al. 2017).

Quality of movement is affected in slow motion (Arzi et al. 2014), pain can be provoked with quick active movement (Gross 1989), and apprehension is seen with quick passive movement (Milgrom et al. 2014) in patients with shoulder instability. Therefore, quantitative analysis of the humeral translation in the healthy shoulder could render clinically important information for dynamic glenohumeral stability. This study could provide some evidence to detect abnormal humeral translation by comparing with normal translation of the humeral head among the different rotation velocities as the humeral head cannot be positioned in the center of the glenoid, especially during higher active rotation velocity in patients with dynamic instability of the shoulder. The hypothesis of this study was that in a normal shoulder, there would be some humeral translation through shoulder rotation although there would be no sudden or marked deviation of the humeral head during active shoulder rotation regardless of motion velocity. The purpose of this study was to clarify translation of the humeral head at different motion velocities during active shoulder rotation with the arm by the side of the body.

## Methods

### Study design and participants

The observational and experimental study was conducted at Nobuhara Hospital in Japan. Ten healthy adults (eight men and two women, ten pairs of shoulders, average age 27.80 ± 6.05 years old) with no current or past history of cervical, thoracic, or shoulder disorder participated in this study. Physical examination of joint instability was done prior to MRI scan and we confirmed that the shoulders were structurally and dynamically stable. They were subject to exclusion if they had general magnetic resonance imaging (MRI) contraindications (Dill 2008).

### Instrumentation

A 0.4 T open MRI scanner (Aperto Eterna, Hitachi Medical Corporation, Japan) was used in this study. Shoulder rotation on an axial plane was captured every 0.5 s with a T2/T1 weighted image. The axial plane in this study was defined as where the scanning plane passed through the maximum width of the glenoid. The following sequence in a modified coherent gradient echo technique using an MRI device was used, with TR at 4.4 ms, TE at 2.2 ms, flip angle at 90°, slice thickness at 1.7 mm, and a bandwidth of 160 kHz. The field of view and matrix were set at 32 cm by 32 cm and 256 by 256 pixels, respectively.

### Procedure

**Fig. 1:**
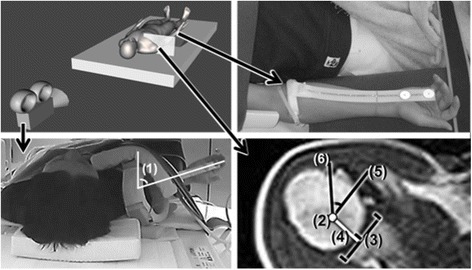
Experimental imaging. Fig. 1a (left top) shows the view of the video camera that was synchronized with the MRI. Fig. 1b (right top) shows a non-constraining brace applied to the subject’s forearm. The position of the brace was not affected by pronation or supination of the forearm. Fig. 1c (left bottom) is a frame captured by the video camera, showing (1) the shoulder rotation angle. Fig. 1d (right  bottom), taken from an MRI scan, shows how translation of the humeral head and rotation angle on MRI are defined. (2) Center of the humeral head. (3) Center of the glenoid fossa. (4) Line from the center of the humeral head perpendicular to the glenoid. Translation of the humeral head is defined as the distance between (3) and (4). (5) Line parallel to a line connecting the anterior and posterior edges of the glenoid and crossing the center of the humeral head. (6) Line connecting the bicipital groove to the center of the humeral head. The rotation angle of the humeral head is defined as the angle between (5) and (6)

The subject was asked to lie supine in an open MRI and to rotate the shoulder with the arm by the side of the body. A 30 degree elbow flexed position was maintained on the wedge shaped stand to enable sliding of the forearm during shoulder rotation (Fig. 1a). This position was determined in a pilot study to avoid the hand of subject hitting the coil of the MRI. Active rotation was controlled by a digital metronome at low (15 cycles/ min), medium (37.5 cycles/ min), and high (52.5 cycles/ min) speeds. The three rotational velocities were determined in a pilot study of measuring maximum (i.e. high velocity) and minimum (i.e. low velocity) angular velocities during active shoulder rotation in patients with minor instability and pain. Medium velocity was defined as the middle of high and low velocities. One shoulder rotational cycle of repetitive shoulder rotational movement in this study was defined as shoulder rotation starting from maximum internal rotation, reaching maximum external rotation, and returning to maximum internal rotation. Each subject was instructed to perform shoulder rotation through maximum active range of motion for 20 s. An examiner measured range of shoulder rotation of each subject beforehand and checked via a video camera monitor whether or not the subject reached the end of internal or external rotation. Images were captured once the subject mastered the technique of the rotation movements required for the three rotation velocities. They were able to do that with a few minutes of practice. This practice was done prior to every shoulder image scanning.

A digital clock, synchronized with the time of capturing open MRI images, was displayed on the screen of an open MRI terminal. The shoulder rotation movements were captured using a digital, full high-definition video camera (resolution 1920 by 1080 pixels) (Fig. 1c). The digital clock on the screen of the open MRI terminal was recorded in the visual field of the monitor camera so that the MRI images and shoulder rotation positions could be synchronized. Neutral rotation for this study was defined as when the forearm reached a full vertical position.

### Data analysis

All imaging data of the MRI and video camera were analyzed after the initial six seconds in order to exclude the transitional period from resting position. The humeral head position and rotation angles on MR imaging and video camera were measured using Matlab 2016b (Mathworks Inc., Massachusetts, USA) before computing the translation of the humeral head. All the data analyses were undertaken by one examiner.

### Center of humeral head on MRI

The center of the humeral head was estimated as the center of a circle fitted to curvature of the head by the least squares method (Fig. 1d). The center of the glenoid was determined by bisecting a line between the anterior and posterior edges of the glenoid. Translation of the humeral head was defined in this study as the distance between the center of the glenoid and the perpendicular intersection at the glenoid of a line from the center of the humeral head (Fig. 1d).

### Shoulder rotation angle on video and MRI

The external rotation angle of the shoulder with the arm by the side of the body was measured as the angle between vertical and the orientation of the forearm (Fig. 1c). A non-constraining brace was put on the forearm to aid in discerning the orientation of the forearm (Fig. 1b). The orientation of the forearm was not affected by pronation or supination when the brace was applied in a pilot study. The rotation angle of the humeral head was the angle formed between a line connecting the bicipital groove to the center of the humeral head and a line parallel to a line connecting the anterior and posterior edges of the glenoid fossa that crossed the center of the humeral head (Fig. 1d). The three angular velocities of rotation were computed.

The range of external and internal rotation with the arm by the side of the body was measured beforehand so that the rotation cycle of the shoulder position for each subject could be computed in terms of *shoulder rotation angle/ maximum rotation angle*. Excursion in one rotation cycle (from full external rotation to full internal rotation and back to full external rotation) was expressed as a percentage. A rotation from 0% to 100% represents external rotation and from 100% to 0% internal rotation. This rotation cycle was used to normalize the slackened capsuloligamentous area in shoulder rotation range. Translation of the humeral head was computed as change in position of the humeral head every 20% of the rotation cycle, using linear interpolation.

### Statistical analysis

An intraclass correlation coefficient (ICC(1, 2)) for the reliability of the examiner measuring rotation angles on the MRI was assessed in which one hundred scans of MRI were randomly selected and analyzed from the MR images of the participants. These MRI scans were analyzed again after a period of one week. Interpretation of ICC was judged according to four grades, poor (less than 0.5), moderate (0.5 to 0.75), good (0.75 to 0.90), and excellent (more than 0.90) (Koo and Li 2016). Pearson’s correlation coefficient was used to assess correspondence of the rotation angles on the MRI and the video camera. Pearson’s correlation coefficient was interpreted on the basis of its magnitude (Mukaka 2012).

The rotation arc at three rotation velocities was compared using one-way analysis of variance. Repeated measures two-way analyses of variance and Tukey-Kramer tests were used to analyze changes in position of the center of the humeral head among three rotational velocities during the rotational cycle. Statistical significance in analysis of variance was set at 0.05.

To judge sample size of this study, we performed a power analysis from our data of maximum translation of the humeral head in low and high velocities using Cohen’s d, since humeral translation at different motion velocities has not previously been investigated in healthy shoulders. Interpretation of Cohen’s d was judged according to three grades: small (0.2), medium (0.5), and large (0.8) (Lakens 2013).

## Results

Reproducibility of the data was excellent, with an ICC of 0.98. Correlations of rotation angles at the three angular velocities appear in Table 1. There were no statistically significant differences of rotation arc among the three rotation velocities (*p* > 0.05). The angular velocities in this study ranged from 36°/s to 117°/s (Table 1). The effect size of this study showed medium power (*d* = 0.50782).

**Table 1:** Parameters of active shoulder rotation at three motion velocities

	Low	Medium	High
Correlation of rotation angles (video and MRI)	0.90	0.84	0.77
External rotation arc (Deg)	53.57 ± 14.85	52.90 ± 11.43	52.43 ± 11.57
Internal rotation arc (Deg)	14.66 ± 11.43	13.76 ± 10.82	12.75 ± 5.77
Rotation arc (Deg)	68.23 ± 17.73	66.66 ± 17.57	65.18 ± 14.76
Angular velocity (°/s)	34.12 ± 8.87	83.33 ± 21.97	114.07 ± 25.84

**Fig. 2:**
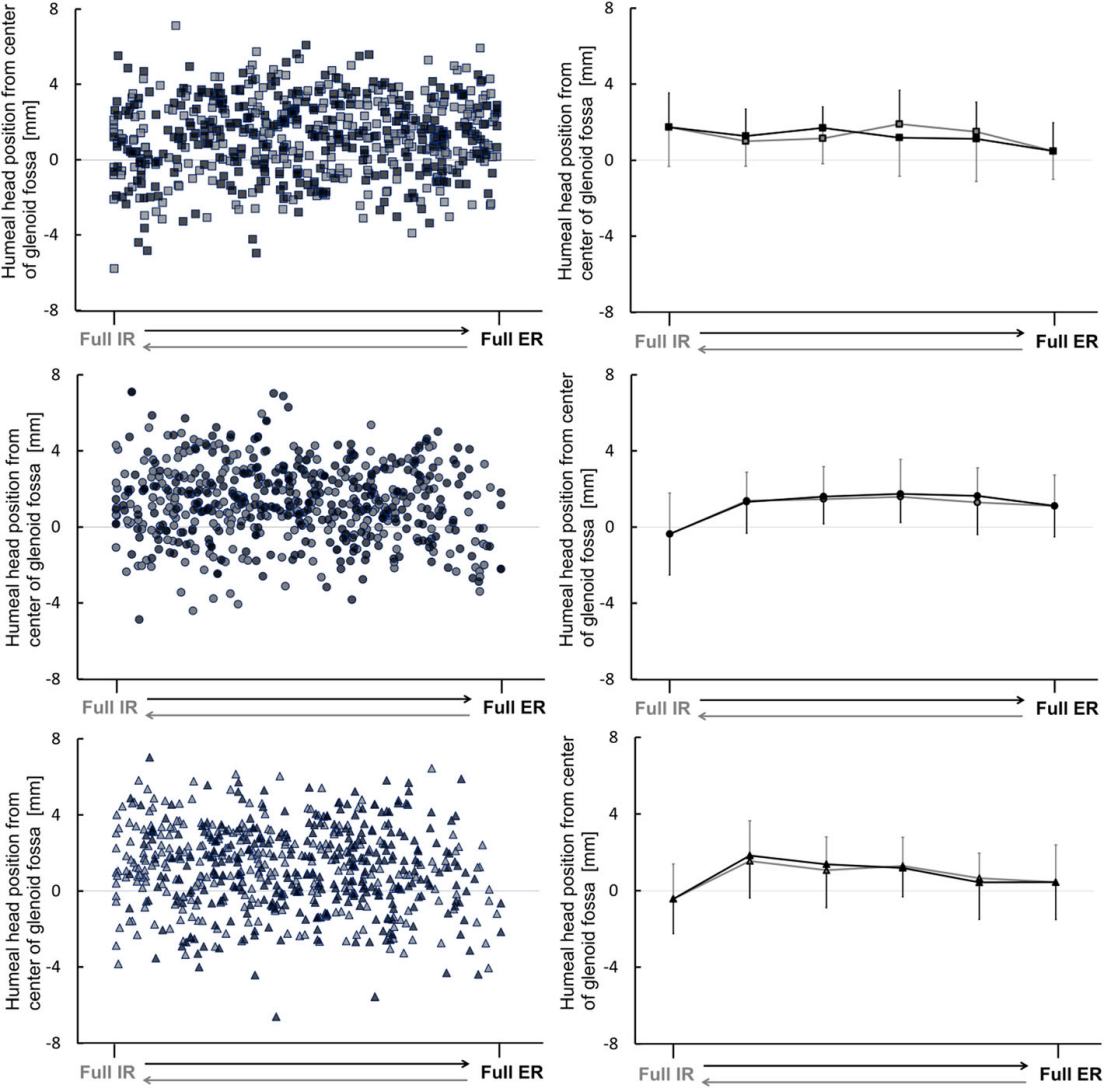
Distribution of humeral head position (the left scatter plot graphs) and mean value of humeral head position (the right line graphs). X-axis represents shoulder rotation (Full IR: Full internal rotation; Full ER: Full external rotation). Y-axis represents the humeral head position from the center of the glenoid fossa (mean ± SD mm). Square markers (top row) are for low velocity. Round markers (middle row) are for medium velocity. Triangular markers (bottom row) are for high velocity. The darker markers and lines represent IR and the lighter markers and lines represent ER

Position of the humeral head center was shown in scatter plot graphs and in line graphs at a given position using the least square methods in the rotation cycle at each of the angular velocities (Fig. 2). The course of humeral translation is shown in Table 2. There were no statistically significant differences of humeral head position or of humeral translation among the three rotation velocities (*p* > 0.05).

**Table 2:** Anterior translation of the humeral head from the center of the glenoid fossa at every 20% of the rotation cycle (mean ± SD mm)

Movement	Position		Low			Medium			High		
ER motion	Far ER	0–20%	0.65	±	1.30	0.74	±	1.92	2.62	±	2.19
	Near ER	20–40%	0.32	±	2.55	0.60	±	2.03	−0.01	±	1.66
	Mid ROM	40–60%	− 0.57	±	1.63	0.05	±	1.78	−0.03	±	1.43
	Near IR	60–80%	−0.21	±	1.24	−0.33	±	1.98	−1.17	±	1.44
	Far IR	80–100%	−0.67	±	2.00	−0.75	±	2.17	0.30	±	0.62
IR motion	Far IR	100–80%	1.44	±	2.45	0.14	±	2.49	0.93	±	1.85
	Near IR	80–60%	0.65	±	1.64	0.31	±	1.42	0.34	±	1.84
	Mid ROM	60–40%	−0.65	±	1.84	0.07	±	1.35	−0.15	±	1.37
	Near ER	40–20%	−0.46	±	1.49	−0.51	±	1.18	0.31	±	2.67
	Far ER	20–0%	0.39	±	1.81	−0.63	±	1.81	−1.51	±	1.60

*IR* Internal Rotation, *ROM* Range of Motion, *ER* External Rotation, *0%* Full IR, *100%* Full ER

## Discussion

This study provides normal values of humeral translation during active shoulder rotation in the healthy glenohumeral joint at different motion velocities. In normal shoulders, subtle changes of humeral head position were observed at different rotational velocities and directions of motion, although there were no statistically significant differences in the position and translation of the humeral head among the three angular velocities. The results of this study demonstrated an aspect of dynamic glenohumeral stability in which that humeral translation was not influenced by slow or quick motion velocities. That is what clinicians have believed from their hands-on findings of dynamically stable joints. The methodology in this study offers visualization of normal intraarticular movement of the glenohumeral joint for comparison with shoulder dysfunction initially detected by the clinician manually. The results of this study provide such reference values of the normal glenohumeral joint.

### Precision of methodology

Shoulder rotation angle of the MRI correlated highly with that of the video camera at all angular velocities. Active rotation range of motion did not statistically differ among the three angular velocities. These findings demonstrate that intraarticular movement was assessed with essentially the same precision of shoulder rotational position and active shoulder range of motion at all three angular velocities. The rotation angle in this study was close to that in previous studies (Bey et al. 2008; Dal Maso et al. 2014). In terms of possible rotation angle difference, our results showed that the value of standard deviation of rotation arc was less than 20°. Even 10% of rotation cycle shows that possible angle difference was 2°. Hence, we judged the differences of rotation angle between each subject were not serious issue since the nature of this difference is close to step length between people.

Intraarticular movement at the three rotational velocities in this study was assessed through a range of motion similar to that of previous studies. These findings further reveal that dynamic glenohumeral instability can be analyzed at angular velocities as high as 114°/s, higher than angular velocities observed in previous studies (Bey et al. 2008; Matsuki et al. 2012; San Juan et al. 2013; Pierrart et al. 2014). Shoulder rotation accompanies elevation movements in day-to-day activities. In activities of daily living it is less than 30°/s (Dal Maso et al. 2015). The methodology of this study may thus lend itself to detecting exceeded translation of the humeral head in the day-to-day activities. Dynamic instability in the glenohumeral joint causes shoulder impingement and pain (Charbonnier et al. 2015)^,^ as well as decreased reproducibility of glenohumeral movements (Barden et al. 2004). Validity of dynamic evaluation of the glenohumeral joint at different rotational speeds needs to be investigated in future work by analyzing unstable joints that have clinical symptoms.

### Position of humeral head

Shift in humeral position from internal to external rotation at low velocity differed from previous studies (Bey et al. 2008; Kozono et al. 2017). The main methodological difference from previous studies (Bey et al. 2008; Kozono et al. 2017) was that shoulder rotation in this study was repetitive shoulder rotation. The humeral head might have positioned itself differently for repetitive continuous shoulder rotation as internal rotation started immediately after external rotation in this study. The humeral head position at low velocity remained anterior to the center of the glenoid fossa, although the subject’s supine position might have been a factor to mechanically translate the humeral head posteriorly. This result implies that the humeral head at low velocity was positioned near the center of the glenoid by appropriate muscle control in conjunction with the mechanical conditions of gravity and inertial force during shoulder rotation.

### Translation of humeral head

Subtle differences between angular velocities were seen during shoulder rotation although our findings imply that the humeral head center was stable in the normal glenohumeral joint regardless of angular velocity. The humeral head translated posteriorly at the end of internal rotation at higher velocities since the scanning position for three angular velocities was the same. At the end of internal rotation, the humeral head was thus situated posteriorly at 0.36 ± 2.15 mm for medium velocity and at 0.43 ± 1.82 mm for high velocity, but anteriorly at 1.75 ± 1.24 mm for low velocity. The results suggest that rotational velocity influences intraarticular movement during ordinary shoulder motions. Consider how the humeral head is subjected to accelerations, decelerations and changes direction of shoulder rotation. This process of decelerating internal rotation prior to initiating acceleration for external rotation resulted in a posterior location of the humeral head at the end of internal rotation. Near the beginning of high-velocity external rotation, the humeral head might be influenced by sudden muscle contraction that first decreases internal rotation velocity and then generates the opposite motion. Changing the direction of rotation involves appropriate muscle activation to decelerate one joint movement and then generate the opposite joint movement while maintaining a stable intraarticular environment. After the late phase of internal rotation, the change in humeral translation was more delayed at high angular velocity than at medium angular velocity. This illustrates how the onset of humeral translation differs among the three angular velocities and provides evidence, based on the healthy glenohumeral joint, for assessing abnormal humeral translation.

At a given angle of rotation, translation during internal rotation differed from that during external rotation, especially if comparisons are made at the beginning of rotation versus at the end of rotation (Table 2). Translation of the humeral head ranged from 0.14 mm anteriorly to 2.62 mm anteriorly in the first 10% of rotation and from 1.51 mm posteriorly to 0.39 mm anteriorly in the last 20% of rotation at the three angular velocities in this study. Our results imply that the direction of rotation influences intraarticular movement at the same position. The anterior translation at the beginning of internal and external rotation might be due to the effect of the agonist muscles and the relatively slack structure of anterior shoulder tissues. The humeral head translates anteriorly at the beginning of internal rotation as contraction by internal rotator muscles pulls it. The anterior translation at the beginning of external rotation might occur as the contraction by external rotator muscles extrudes the humeral head. In a cadaveric study, this phenomenon was attributed to capsular tightness on the contralateral side (Harryman et al. 1990). During active shoulder rotation, extrusion of the humeral head is thus likely toward the side contralateral to that of the contracting muscles. The degree of normal intraarticular movement thus depends on the rotation angle (Matsuhashi et al. 2013). Assessing intraarticular movement during active movement at various motion velocities is important for detecting the effect of motor control.

### Advantage of methodology in this study

An advantage of MR scanning for the assessment of the humeral translation in this study is the non-invasive nature of the instrument and no need to prepare for the analysis. In a fluoroscopic studies (Matsuki et al. 2012; San Juan et al. 2013), a biplane radiographic study (Bey et al. 2008) and a motion capture study (Dal Maso et al. 2015), the number of subjects was restricted due to radiation exposure (Bey et al. 2008; Matsuki et al. 2012; San Juan et al. 2013) or to markers mounted into bone (Dal Maso et al. 2014, 2015). A three-dimensional (3D) MRI study attempted to scan continuous shoulder elevation (Pierrart et al. 2014). The scanning time for one position requires 4 s in the 3D MRI study (Pierrart et al. 2014). Although cine MRI contains less information than 3D MRI, it can scan the shoulder at higher velocity. This study provides data on normal intraarticular movement of the glenohumeral joint divided into 5 phases of one shoulder rotation cycle at various rotation velocities. The methodology in this study might be used to visualize shoulder dysfunction subsequent to manual detection. Our findings thus might be useful for detecting, during rotary movement at different rotation velocities, abnormal humeral translation due to macro or micro trauma.

### Limitation of this study

A limitation of this study was that the motion analyzed was restricted to a single rotatory plane. This is a far cry from continuous functional shoulder movements such as combing hair or reaching for an object. Future research is required to confirm the role of varying shoulder rotational speed in analyzing dynamic instability of the glenohumeral joint to achieve enhanced performance of activity in the shoulder.

## Conclusions

Intraarticular movement of the glenohumeral joint was validated at different rotation velocities using cine MRI. Our results revealed that, in the dynamically stable glenohumeral joint, humeral head position and translation did not significantly differ among three rotation velocities of active shoulder rotation, although subtle changes of humeral head position were observed at different rotational velocities and directions of motion. The accompanying changes of humeral head translation might result from inertial forces and muscle contraction during motion.

## Abbreviation

3D: Three dimensional
ER: External Rotation
ICC: Intraclass correlation coefficient
IR: Internal Rotation
MRI: Magnetic Resonance Imaging
ROM: Range of Motion
